# Role of the C-terminal domain of the HIV-1 glycoprotein in cell-to-cell viral transmission between T lymphocytes

**DOI:** 10.1186/1742-4690-7-43

**Published:** 2010-05-12

**Authors:** Vanessa Emerson, Claudia Haller, Tanya Pfeiffer, Oliver T Fackler, Valerie Bosch

**Affiliations:** 1 Forschungsschwerpunkt Infektion und Krebs, F020, Deutsches Krebsforschungszentrum, Im Neuenheimer Feld 280, 69120 Heidelberg, Germany; 2 Department für Infektiologie, Virologie, Universitätsklinikum Heidelberg, Im Neuenheimer Feld 324, 69120 Heidelberg, Germany

## Abstract

**Background:**

Mutant HIV (HIV-Env-Tr712) lacking the cytoplasmic tail of the viral glycoprotein (Env-CT) exhibits a cell-type specific replication phenotype such that replicative spread occurs in some T-cell lines (referred to as permissive cells) but fails to do so in most T-cell lines or in PBMCs (referred to as non-permissive cells). We aim to gain insight on the underlying requirement for the Env-CT for viral spread in non-permissive cells.

**Results:**

We established that in comparison to HIV-Wt, both cell-free and cell-to-cell transmission of mutant HIV-Env-Tr712 from non-permissive cells were severely impaired under naturally low infection conditions. This requirement for Env-CT could be largely overcome by using saturating amounts of virus for infection. We further observed that in permissive cells, which supported both routes of mutant virus transmission, viral gene expression levels, Gag processing and particle release were inherently higher than in non-permissive cells, a factor which may be significantly contributing to their permissivity phenotype. Additionally, and correlating with viral transfer efficiencies in these cell types, HIV-Gag accumulation at the virological synapse (VS) was reduced to background levels in the absence of the Env-CT in conjugates of non-permissive cells but not in permissive cells.

**Conclusions:**

During natural infection conditions, the HIV-Env-CT is critically required for viral transmission in cultures of non-permissive cells by both cell-free and cell-to-cell routes and is instrumental for Gag accumulation to the VS. The requirement of the Env-CT for these related processes is abrogated in permissive cells, which exhibit higher HIV gene expression levels.

## Background

Infectious spread of viruses to new target cells *in vitro *and *in vivo *occurs either via infection with released cell-free virions or by direct transmission of virions from cell to cell. Some viruses e.g. human T-cell leukemia virus type 1 (HTLV-1) or Spuma retroviruses employ solely the cell-to-cell route and cell-free viral infection is negligible [1]. In the case of human immunodeficiency virus type 1 (HIV-1), both routes of viral spread are possible, but already very early reports documented that transmission by the cell-to-cell route was far more efficient [2-4]. A series of more recent studies have now established cell-to-cell transmission as the predominant mode of HIV-1 spread in T lymphocyte cultures [5-9]. Analogous to the situation with HTLV-1 [10], confocal microscopic analyses of infected T lymphocyte cultures revealed close conjugates of infected donor cells and uninfected target cells and cell-to-cell transmission of virus particles across the cell contact site referred to as the virological synapse (VS). In addition, several types of membrane bridges have also been observed to mediate transport and infection of HIV-1 particles between T lymphocytes [11,12]. The term cell-to-cell transmission thus summarizes all types of HIV-1 spread between physically connected infected donor and uninfected target cells, including spread via short distance transmission of cell-free virions and directional transport along cellular protrusions [13]. Although the relative contribution of these transmission modes still remains to be determined, accumulation of both cellular and viral proteins at these cell contacts has been established as a hallmark of efficient HIV-1 cell-to-cell spread. Such polarisation includes accumulation of the viral structural proteins Gag and Env as well as the microtubule organising centre (MTOC) at the donor cell contact, while cellular receptors (CD4, coreceptor) and cytoskeletal proteins (F-actin, talin) typically accumulate at the target cell contact [12,14-18]. Even though some host cell signalling cascades that govern polarisation of HIV-1 Gag to the VS have been identified [18], it remains unclear which domains of viral Env and Gag proteins are operational in mediating transport and functional accumulation of HIV-1 structural proteins to the cell contact site.

The HIV-1 glycoprotein carries a very long cytoplasmic C-terminal tail (CT, 151 amino acids (aa) long) which is absolutely required for replication *in vivo*. Mutant virions lacking this region exhibit a cell-type dependent phenotype *in vitro *such that replicative virus spread occurs in some cell lines (termed permissive cells, e.g. MT-4 cells) but not in the majority of T-cell lines (termed non-permissive cells, e.g. H9 cells) nor in PBMCs [19-22]. The basis for the requirement for the Env-CT for viral spread in non-permissive cells, and the reason(s) underlying the permissivity phenotypes of different T-cell lines are presently unclear and are the focus of this study.

Genetic [23,24] and protein association data [25,26] support the view that there is a functional interaction between the Env-CT and the viral matrix protein (MA). This interaction appears to be involved in a number of processes. Thus, in released immature virions, Env-CT interaction with the unprocessed Gag precursor prevents premature fusion activity of Env [27]. The Env-CT domain has also been shown to impact intracellular localisation of Gag and the subcellular localisation of particle assembly. In the absence of Wt-Env, HIV particle release from polarised epithelial cells was shown to occur at both apical and basolateral membrane surfaces, yet in its presence release occurred exclusively at the basolateral membrane [28,29]. The Env-CT domain and in particular a membrane-proximal tyrosine-based signal within it were shown to be instrumental in this. Furthermore, removal of the same Env-CT tyrosine-based signal has been reported to inhibit polarised budding of HIV in T-lymphocytes and to reduce cell-to-cell viral transmission [30]. A further event, which for many years has been discussed to involve the Env-CT and its interaction with Gag, concerns Env incorporation into released virions. Nevertheless, HIV-Env-Tr712 virions, encoding Env lacking the CT domain, when produced in transfected adherent cells or in infected permissive cells did incorporate truncated glycoprotein and were infectious [20,31,32]. On the other hand, it was reported several years ago that cell-free mutant HIV-Env-Tr712 virions, released from non-permissive cells, were non-infectious and that this correlated with a lack of mutant glycoprotein incorporation [19,21]. In a previous study, aimed at further studying the defective phenotype of HIV-Env-Tr712, we had also analysed the infectivities of cell-free mutant virus particles. These were generated by efficiently infecting non-permissive H9 producer cells with VSV-G pseudotyped derivatives and collecting the newly generated (unpseudotyped) virions 48 h later. However, under these experimental conditions, cell-free HIV-Env-Tr712 virions were only marginally reduced in their infectivity [33,34], an observation which was difficult to reconcile with the total lack of spread of mutant virus in these cells. The reason for these discordant observations has remained unclear until now. In this report, we considered the possibility that, in contrast to cell-free infectivity, cell-to-cell virus transmission of HIV-Env-Tr712 in non-permissive T-cells could be more severely impacted and that this could be the reason for the block in viral spread.

## Methods

### Constructs

Proviral plasmids were based on pNL4-3 ^BH10 env ^(referred to here as pNL-Wt) [35]. pNL-Env-Tr712 encodes Env with a stop codon at position 713, i.e. lacking 144 aa [32]. pNL-Env^Fus- ^is fusion-defective due to exchange of the second aa (V513E) within the fusion peptide of gp41 [36] and pNL-ΔEnv fails to synthesise Env due to an introduced frame-shift mutation [37]. pMD.G is an expression vector for the G glycoprotein of vesicular stomatis virus (VSV) [38].

### Cell lines, transfections, analysis of cell-free virion infectivities

293T cells were cultivated in DMEM medium, 10% foetal calf serum (FCS) and all T-cell lines, namely MT-4, MT-2, C8166, H9, CEM-SS and Jurkat cells, in RPM-I medium, 10% FCS. H9 cells constitutively expressing GFP (H9-GFP) have been previously described [39]. Procedures for the infection of T-cells with VSV-G pseudotyped virions leading to the generation of T-cell-produced cell-free progeny virions have been previously described [33,34]. Briefly, VSV-G pseudotypes, released into the supernatant of 293T cells co-transfected with proviral pNL4-3 ^BH10 env ^plasmids [32] and pMD.G, were quantified by HIV-CA ELISA (Innogenetics, Belgium) and employed to infect fresh MT-4 or H9 T-cells. At 5 h p.i., input virions were removed,;the cells were washed three times with medium and then further incubated for 43 h. In initial experiments, we aimed to efficiently infect T-cells and thus employed saturating amounts of 293T cell supernatants containing VSV-G pseudotyped viruses (15-25 μg virus-associated p24 per 10^6 ^cells, infection level 50-100%) [33,34]. In later experiments, limiting amounts of supernatants (0.5-3 μg virus-associated p24 per 10^6 ^cells), which resulted in only a fraction of the cells (<20%) becoming infected, were employed. At 48 h p.i., the numbers of infected cells were quantified by intracellular p24 FACS using PE-labeled HIV-p24 antibody (KC57-RD1 from Coulter, Florida) and the single-round infectivities of newly produced cell-free virions in the culture supernatants were assessed in MT-4 target cells as described previously [33,34]. MT-4 target cells allow efficient cell-free infection i.e. a high percentage (up to 100%) of the cells becomes infected. This allows better discrimination between the infectivities of different viruses than in H9 cells in which cell-free infection with the same amounts of virions results in only a small percentage (<5%) of the cells initially becoming infected [33].

### Cell-to-cell viral transmission

In addition to analysis of the cell-free infectivities of released virions, at 48 h p.i. the abilities of the infected cells to transmit virus to new target cells by the cell-to-cell route were assessed. Stable GFP expressing H9 cells (H9-GFP) or dye-loaded MT-4 cells were employed as targets. Dye loading of MT-4 cells was achieved by incubation with 10 μM CellTracker Green CMFDA (Molecular Probes, Eugene, USA) in RPM-I medium without additives for 30 min at 37°C. In an initial protocol, donor cells were efficiently infected with saturating amounts of VSV-G pseudotyped virus (equivalent to 15-25 μg virus-associated p24 per 10^6 ^cells). The number of infected donor T-cells was determined by intracellular p24 FACS (and was >50%) and then adjusted to 50% with uninfected cells. These infected cells were then mixed and incubated with a 4-fold excess of labelled target cells i.e. there were 10% infected donor cells present in the coculture containing 4 × 10^6^cells in a volume of 10 ml. In later experiments, donor T-cells were infected with limiting amounts of VSV-G pseudotyped virus (equivalent to 0.5-3 μg virus-associated p24 per 10^6 ^cells). The number of infected cells, as determined by intracellular p24 FACS, was in the range of 10-20% and was adjusted to 10% with unifected cells and then mixed and incubated with a 9-fold excess of labelled target cells i.e. there were 1% infected donor cells present in the coculture containing 4 × 10^6 ^cells in a volume of 10 ml. 5 h post-mixing, the CXCR4 antagonist, AMD3100 (1 μg/ml), was added to the mixtures and 43 h later, the percentage of labelled target cells infected, and thus expressing HIV-CA, was established by intracellular p24 FACS (10,000 gated cells were analysed in each case). The value obtained for pNL-Wt was set at 100% and the values for pNL-Tr712 and pNL-Env^Fus- ^calculated relative to this.

### HIV gene expression in permissive and non-permissive T-cell lines

Non-permissive or permissive T-cells were infected with limiting amounts of VSV-G pseudotyped HIV-Wt or HIV-Env-Tr712 virions (0.5-3 μg virus-associated p24 per 10^6 ^cells resulting in 10-20% infection level). At 5 h p. i., input virions were removed, and cells washed three times with medium before being further cultivated in fresh medium containing 1 μg/ml AMD 3100 to prevent viral spread. At 48 h p. i., an aliquot of the infected cells was subjected to intracellular p24 FACS to establish the percentage infected cells. The total cell densities and the percentages of cells infected in the respective cultures were equalised by addition of uninfected cells and culture medium. Lysates of equal numbers of cells, now containing equal percentages of infected cells, were prepared, and protein determination using standard procedures showed that these did not differ significantly between the different T-cell lines. Equal aliquots were subjected to Western blot analyses employing anti-p24 mAb 183-H12-5C [40], anti-tubulin mAb (Sigma) and rabbit anti-gp120 serum. Comparative densitometric quantitation of specific bands on different exposures of the blots to film was carried out using the Image J software from the NIH. The amounts of virions released into the respective culture supernatants were determined by HIV-CA ELISA (Innogenetics, Belgium).

### Env incorporation into virus particles

HIV-Wt and HIV-Env-Tr712 virions were enriched from culture supernatants of H9 (np) cells, weakly infected (< 20%) as described above. At 48 h p.i., supernatants were filtered (0.450 μm filter) and subjected to ultracentrifugation through a 20% sucrose cushion in PBS. Equal amounts of pelleted virions (as determined by HIV-CA ELISA) were subjected to Western blot analysis employing rabbit anti-gp120 serum, gp41 mAb Chessie 8 [41] or anti-p24 mAb 183-H12-5C [40]. Densitometric quantitation of specific bands was carried out as above. The amount of gp120 normalised to p24 amount was set at 100% for HIV-Wt and the relative gp120 incorporation into HIV-Env-Tr712 virions calculated relative to this.

### HIV-Gag localisation in conjugates between infected and non-infected cells

In order to identify newly formed cell conjugates, uninfected target cells were labelled with 5 μM CellTracker Blue CMAC (Molecular Probes, Eugene, USA) prior to mixing. For this, cells were incubated in 5 μM dye in RPM-I medium without additives for 30 min at 37°C. Infected donor H9 or MT-4 cells were prepared by infection with VSV-G pseudotyped Wt or mutant HIV (see above). For conjugate formation 2.5 × 10^5 ^infected cells were mixed with 2.5 × 10^5 ^uninfected labelled target cells in a volume of 100 μl RPM-I, 1% FCS in an Eppendorf tube, centrifuged for 5 minutes at 200 g and incubated for 15 min at 37°C. The cell mixture was then gently resuspended and transferred onto poly-L-lysine coated cover-slips. For poly-L-lysine coating, cover-slips were cleaned with 1 M HCl/70% ethanol for 30 min, dried at 60°C for 30 min, treated with 0.01% poly-L-lysine solution for 10 min and dried again at 60°C for 30 min before adding PBS for storage. Once added to the coated cover-slips, cells were incubated for a further 10 min at 37°C, and cells attached to the cover-slips were fixed with 3% paraformaldehyde in PBS for 1.5 h. HIV-Gag was stained with rabbit-anti-CA [42] and cellular actin was stained with TRITC-labelled phalloidin, cover-slips were mounted in Elvanol and analyzed with a Leitz DMRBE fluorescence microscope (Leica, Germany) using a 100× oil immersion objective. The localisation phenotypes of actin and HIV-Gag were evaluated and quantitated by three different investigators, two of whom were not aware of the nature of the different samples. Images were taken at a LSM 510 confocal laser-scanning microscope (Zeiss) using a 100× oil immersion objective and processed by Adobe Photoshop.

## Results and Discussion

### Transmission of HIV with C-terminally truncated Env

**Figure 1:**
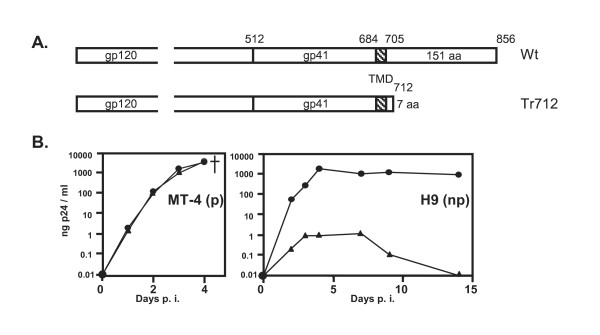
**Replicative spread of HIV-Wt and HIV-Env-Tr712 in different T-cells**. A. Schematic depiction of the Env proteins from HIV-Wt and HIV-Env-Tr712 B. Replication kinetics of HIV-Wt (circles) and HIV-Env-Tr712 (triangles) in MT-4 cells (p) and H9 cells (np) below. Cells were infected with equal amounts of virus (equivalent to 100 ng p24 per 10^6 ^cells) and washed at 5 h p.i. Newly produced virions released into the culture supernatants at the times indicated were quantified by CA-ELISA. Note that at 3-4 d post infection, MT-4 cells were infected to 100% with both viruses (as established by indirect immunofluorescence) and succumbed to HIV induced cytotoxicity. Infection of H9 cells with HIV-Wt reached 100% at 4-5 d post-infection whereas infection with HIV-Env-Tr712 virions (produced in permissive 293T cells) resulted in initial infection of only a low percentage (< 5%) of cells which subsequently vanished from the culture. The cut-off of the assay lies at 0.01 ng/ml.

Fig 1A schematically depicts the Env proteins of HIV-Wt and HIV-Env-Tr712. As detailed in the Introduction and shown in Fig. 1B, HIV-Env-Tr712 virions exhibit a cell-type specific defect such that replicative spread occurs in some T-cell lines (here MT-4 cells, termed permissive (p) cells) but fails to occur in the majority of T-cell lines (here H9 cells, referred to as non-permissive (np) cells). In this report, our emphasis was to analyse the possible impact of Env-CT truncation on cell-to-cell viral transmission in both H9 (np) and MT-4 (p) cells. Infected donor cells were generated by infection with VSV-G pseudotyped Wt or mutant viruses and, in the course of our studies, we observed that their initial infection level markedly influenced experimental outcome. Thus, in this report, we describe cell-to-cell transmission experiments employing highly or weakly infected donor cells and have also analysed cell-free viral infectivities in the context of both of these scenarios.

**Figure 2:**
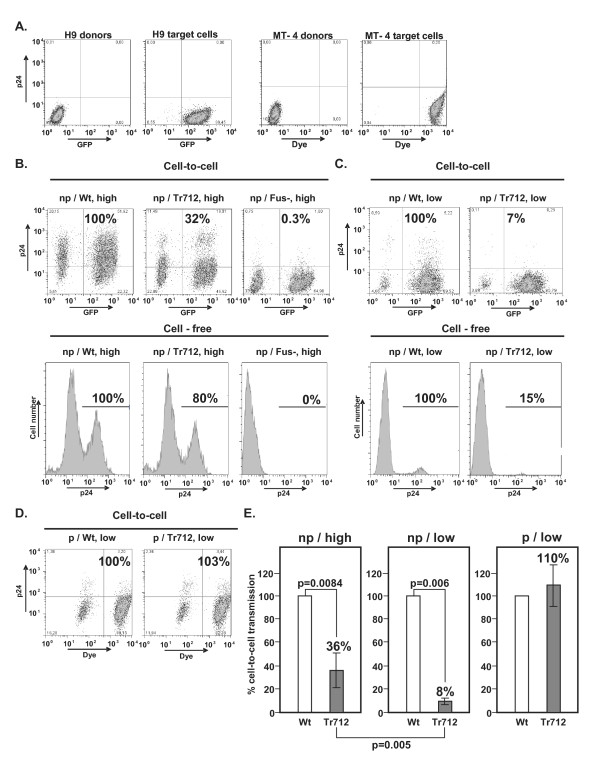
**Cell-to-cell and cell-free transmission of HIV-Wt and mutant virions**. A. FACS analysis of uninfected donor cells and GFP-labelled H9 (np) or dye-labelled MT-4 (p) uninfected target cells. B. Virus transmissions from H9 (np) donor cells highly infected (high) with VSV-G pseudotyped HIV-Wt, HIV-Env-Tr712 or HIV-Env^Fus-^. Top panels: cell-to-cell transmission. Washed donor cells were adjusted to 50% infection level with uninfected cells and then mixed with a 4-fold excess of H9 target cells. FACS analysis was performed as in A. The percentage target cells infected with HIV-Wt was set at 100% and the levels of transmission of HIV-Env-Tr712 or HIV-Env^Fus-^, calculated relative to this. Bottom panels: cell-free infection. Equal amounts of released virions from highly infected donor cells were employed to infect susceptible MT-4 cells as described in the Materials and Methods section. At 48 h p.i., the cells were analysed by intracellular p24 FACS. The percentage of cells infected by HIV-Wt (right peak) was set at 100% and the infectivities of HIV-Env-Tr712 and control HIV-Env ^Fus- ^calculated relative to this. C. Virus transmissions from H9 (np) donor cells weakly infected (low) with VSV-G pseudotyped HIV-Wt or HIV-Env-Tr712. Washed donor cells were adjusted to 10% infection level with uninfected cells and then mixed with a 9-fold excess of H9 target cells. Further procedures were as in B. D. As in C. except that MT-4 cells (p) were employed both as donor and target cells. E. Mean percentage transmission levels, relative to that of HIV-Wt, of HIV-Env-Tr712 from H9 (np) donor cells infected to high levels (left panel) (12 experiments), H9 (np) donor cells infected to low levels (middle panel) (4 experiments) or MT-4 (p) donor cells infected to low levels (right panel) (4 experiments). The statistical significance of the respective differences is shown (Student's t-test).

Donor cells were infected with either saturating or limiting amounts (on average 20 times less than used for saturating infection, see Methods section) of VSV-G pseudotyped HIV-Wt, HIV-Env-Tr712 or, as negative control, HIV-Env^Fus- ^virions. Input pseudotyped virions were thoroughly removed at 5 h p.i.; and 43 h later, infected cells were employed as donors for viral transmission to target cells constitutively expressing GFP (H9 cells) or labelled with a green dye (MT-4 cells). As shown in Fig. 2A, labelled target cells could clearly be distinguished from unlabelled donor cells by FACS analysis. Donor cells infected with saturating amounts of VSV-G pseudotyped virions, and thus highly infected (50-90% level), were adjusted to 50% infection level with uninfected H9 donor cells and then mixed with a 4-fold excess of uninfected labelled target cells (i.e. 10% of the cells in the mixture were infected) (Fig. 2B). Donor cells infected with only limiting amounts of VSV-G pseudotyped virus, and thus weakly infected, (less than 20% infection) were adjusted to 10% infection level and mixed with a 9-fold excess of target cells, (i.e. 1% of the cells in the mixture were infected) (Figs. 2C, D). Five hours post-mixing, the CXCR4 antagonist, AMD3100, was added to the mixture and 43 h later, the number of labelled target cells expressing HIV-CA and thus being productively infected, was established by intracellular p24 staining. In line with previous reports [7,43], we confirmed that addition of AMD3100 prior to mixing of infected and uninfected cells completely inhibited p24 detection in target cells. This means that the assay is not measuring endocytosis of virus particles, but rather only productive viral transmission in conjugates, formed within the 5 h incubation prior to drug addition. Lack of significant detection of transferred "input" virus in the assay is also supported by the fact that, when analysed at early time points post-mixing (e.g. at 6 h), intracellular p24 staining of target cells was negligible.

The fraction of target cells infected by HIV-Wt was set at 100% and, relative to this, the transmission efficiencies of HIV-Env-Tr712 or HIV-Env^Fus- ^were calculated (Fig. 2B, C, upper panels). Additionally, newly synthesised virions in the media of the originally infected H9 T-cells at 48 h p.i. were collected and their infectivities analysed employing susceptible MT-4 cells as targets (Fig. 2B, C, lower panels).

Examples of cell-to-cell and cell-free viral transmission experiments from either highly (Fig. 2B) or weakly (Fig. 2C) infected H9 (np) donor cells or cell-to-cell transmission from weakly infected MT-4 (p) cells (Fig 2D) are shown. The respective mean percentage levels of cell-to-cell transmission of HIV-Env-Tr712 in comparison to HIV-Wt from several experiments employing these different experimental set-ups are shown in Fig. 2E. In line with our previous report [33], when H9 (np) donor cells were highly infected, HIV-Env-Tr712 cell-free virions exhibited only moderately reduced infectivity in comparison to HIV-Wt (to 80%, Fig. 2B, lower panels). Cell-to-cell transmission was somewhat more affected, but this reduction (on average to 36% of HIV-Wt, (Fig. 2B, upper panels, Fig. 2E)) was still relatively moderate considering the total lack of productive viral spread of mutant virions in H9 T-cells (Fig. 1B). As to be expected, transmission of HIV-Env^Fus- ^by both cell-free or cell-to-cell routes was only at background levels.

In contrast to the situation in which the donor cells were highly infected with saturating amounts of VSV-G pseudotypes, when the H9 (np) donor cells were only weakly infected (employing on average 20-fold less VSV-G pseudotypes), the reductions in HIV-Env-Tr712 transmission by both the cell-to-cell route (Fig. 2C, upper panels to 7% of HIV-Wt, mean 8% (Fig. 2E)) or by cell-free virus (Fig. 2C, lower panels to 15% of HIV-Wt) were markedly more pronounced. Under these conditions, these impairments together probably completely account for the observed abrogated spread of HIV-Env-Tr712 in H9 (np) cells (Fig. 1B). Finally, in congruence with their ability to support replicative spread of mutant virus, when permissive donor cells were employed, regardless of their infection level (Figs. 2D, E and not shown), cell-to-cell transmission of HIV-Env-Tr712 was not reduced. Note that in Fig. 2D, due to the low percentage of infected donors (1%), transfer efficiencies of both viruses are low despite both donor and target cells being permissive.

In summary, the results obtained indicate that under low infection conditions of non-permissive cells, which likely reflect the situation in natural infection, the Env-CT appears to play a pivotal role in both cell-free and cell-to-cell infection routes. It is plausible that these two phenotypes may be at least partially related and that reduced infectivity of HIV-Env-Tr712 particles, released locally into the cleft of the VS, may contribute significantly to defective cell-to-cell spread.

**Figure 3:**
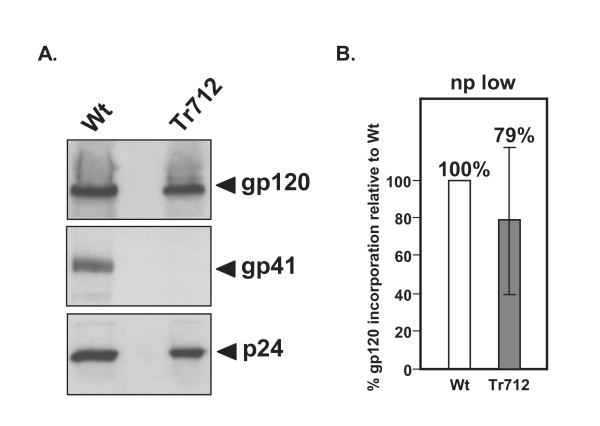
**Env incorporation into Wt-HIV and HIV-Env-Tr712 virion**. Producer H9 (np) cells had been weakly infected (less than 20% infection level). A. Western blot: the top part of the filter has been probed with gp120 antibodies, the middle part with gp41 mAb, Chessie 8 against the Env C-terminal tail (truncated in HIV-Env-Tr712) and the bottom part with p24 mAb. B. Average gp120 incorporation into HIV-Env-Tr712 virions in comparison to HIV-Wt (from 3 independent experiments: individual values 37%, 118%, 83%).

Additionally, it is likely that differences in infection levels of producer cells also account for the fact that, in contrast to our earlier report [33], others had previously reported reduction in the cell-free infectivity of HIV-Env-Tr712 virions produced in non-permissive cells [19,21]. In these latter studies, reduced infectivity had been reported to correlate with a defect in Env-Tr712 incorporation [19,21]. Thus, in order to study this here, HIV-Wt and HIV-Env-Tr712 virions were produced in weakly infected H9 (np) cells (less than 20% infected) and their protein content analysed by Western blot. Three independent experiments were evaluated by quantifying the amount of incorporated gp120 relative to viral p24 content for each virus preparation. As seen in Fig. 3A, gp120 incorporation into HIV-Env-Tr712 virions can clearly be seen and, as shown in Fig. 3B, the amount is, on average 79% of that in HIV-Wt. The reason for this discrepancy between these Env incorporation results and those previously published is presently not known. At any rate, this modest reduction in Env incorporation appears unlikely to account for the strongly reduced infectivity of cell-free HIV-Env-Tr712 virions.

### HIV gene expression in non-permissive and permissive cells

**Figure 4:**
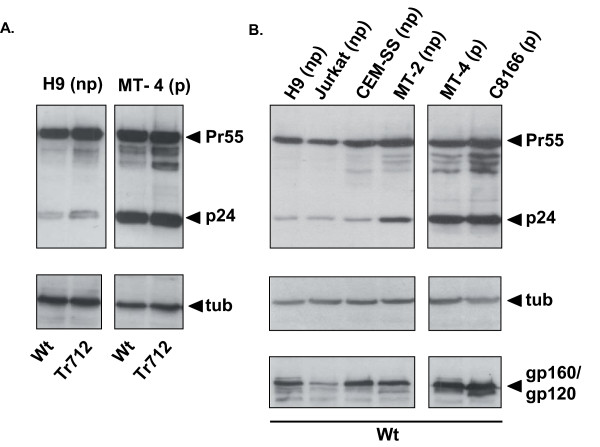
**HIV *gag *gene expression in non-permissive (np) and permissive (p) cells**. A. Western blot analysis employing antibodies to HIV-CA and cellular tubulin of equal amounts of lysates of the indicated cell lines infected to equal levels with HIV-Wt or HIV-Env-Tr712. In this experiment, the amounts of virus released into the culture supernatants (determined by HIV-CA ELISA) were 14 ng/ml and 9 ng/ml for HIV-Wt and HIV-Env-Tr712, respectively, produced in H9 (np) cells and 43 ng/ml for both viruses produced in MT-4 (p) cells. B. Western blot analysis as in A of the indicated cell lines infected to equal levels with HIV-Wt. The positions of the detected gp120/gp160, Pr55^gag^, p24 and cellular tubulin proteins are given on the right.

A plausible explanation for the partial "masking" of the defective phenotype when H9 (np) donor cells are infected at saturating levels with HIV-Env-Tr712 could be that multiple integrated proviruses result in increased HIV gene expression in the producer cell and by this mechanism compensate for the Env-CT truncation. This led to the idea that differences in viral gene expression levels could be an underlying phenomenon contributing to the differences in permissivity of H9 and MT-4 cells. To test this, H9 (np) cells and MT-4 (p) cells were weakly infected with limiting amounts of VSV-G pseudotyped HIV-Wt or HIV-Env-Tr712 virions in principle as reported for Fig. 2. Forty-three hours later, the percentage of infected cells was determined by p24 FACS (these were in the range of 10-20%) and the cultures adjusted (with uninfected cells and medium) to equal total cell densities of equally infected cells. That is, at the time of harvest, the same number of equally infected cells was present in each culture (see also Methods). As shown in Fig. 4A, Western blot analysis revealed that there was a striking difference in the HIV Gag protein profiles in cell lysates of the respective H9 (np) and MT-4 (p) cultures independent of infection being with HIV-Wt or HIV-Env-Tr712. Proteolytic processing of Pr55^gag ^to p24 (CA) was clearly more efficient in MT-4 (p) cells. In these cells about 50% of the total p24-reactive protein was present as p24 monomer while this was only about 15% in H9 (np) cells. Moreover, total amounts of Gag protein, i.e. Pr55^gag^+p24, in MT-4 cells were about twice as much as in H9 cells (Fig 4A). In accordance with this, at comparable levels of infected cells, MT-4 (p) cells had released on average 2-3 times more virus into the supernatant as compared to H9 (np) cells (5 experiments performed). In order to rule out that these observations were restricted to these two T-cell lines, we have analysed a small panel of non-permissive cells, namely H9, Jurkat, CEM-SS and MT-2 cells and, in addition to MT-4 cells, the only further permissive T-cell line known to us, namely C8166 cells. We (not shown) and others [19,21] have confirmed the permissivity status of these cell lines with respect to replication of HIV-Env-Tr712 virus. As shown in Fig. 4B, in all of the non-permissive cells, total Gag and, additionally, gp120/gp160 amounts were lower. Pr55^gag ^processing in the non-permissive cells was less efficient than in both the permissive MT-4 and C8166 cell lines. The effect was less pronounced in the case of non-permissive MT-2 cells but Gag processing was still about 50% of that observed in MT-4 (p) and C8166 (p) cells. However, in the cases of Jurkat (np) and CEM-SS (np) cells, the observed decreases were as pronounced as in H9 (np) cells. Increased Gag expression in permissive cells presumably leads to increased virus particle assembly and release which is the stage at which Gag proteolytic processing occurs. Thus, although it cannot be ruled out that specific cellular environments may affect Gag processing efficiency *per se*, it is rather more likely that increased Gag processing is a direct consequence of higher Gag expression in permissive cells. In addition to the two permissive T-cell lines employed here, several frequently employed adherent cell lines e.g. 293T or HeLa, can be regarded as being permissive inasmuch as HIV-Env-Tr712 virions, produced after transient transfection of proviral DNA, exhibit Wt levels of infectivity. This is again likely to be a consequence of high HIV gene expression at the single cell level presumably overriding the requirement for the Env-CT.

The basis for the observed increased Gag expression in permissive MT-4 and C8166 is presently unknown. Conceivably the HTLV-1 transformation status, and expression of Tax transactivator protein, in both of these cell lines [44,45] may contribute to higher transcriptional activity from the HIV-LTR. However, MT-2 cells are also HTLV-1 transformed and express Tax [45], but are non-permissive for spread of HIV-Env-Tr712. Perhaps in this case, the observed less marked increase in gene expression is not sufficient to compensate for the Env-CT truncation, or additional cellular factors underlying the permissivity phenotype have to be invoked.

We had postulated that in non-permissive H9 donor cells, infected with saturating amounts of VSV-G pseudotyped virions, the requirement for the Env-CT was partially overcome due to enhanced overall gene expression from multiple proviruses. Direct examination of HIV protein amounts per infected cell did reveal a moderate increase (about 20% more CA protein) when H9 (np) cells were infected with saturating, rather than limiting, amounts of HIV-Wt or HIV-Env-Tr712 virions (data not shown).

### CA distribution in cell-to-cell transmission conjugates of T lymphocytes infected with HIV-Wt and mutant HIV

**Figure 5:**
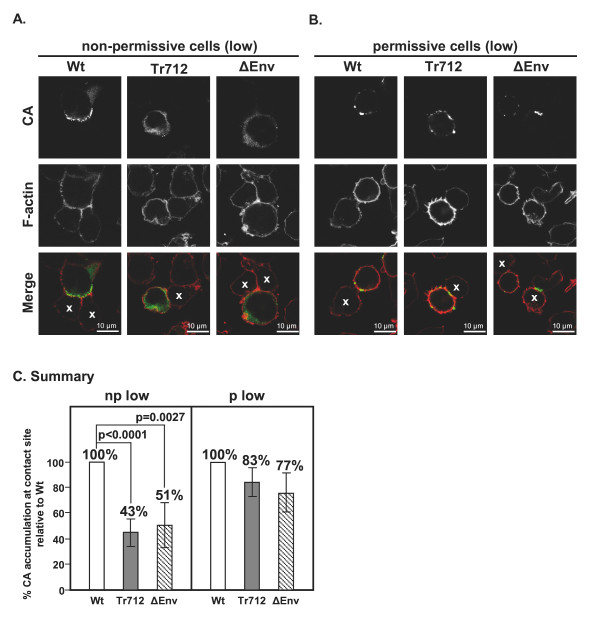
**Analysis of CA accumulation at the VS**. Confocal microscopic analysis of CA and F-actin localisation phenotypes in conjugates of non-permissive H9 cells or permissive MT-4 cells infected with HIV-Wt, HIV-Env-Tr712 or HIV-ΔEnv. Conjugates between infected donor cells and freshly added dye-labelled target cells were generated as described in the Material and Methods section and selected for analysis initially by widefield microscopy. A. Predominant CA localisation patterns in conjugates of H9 (np) cells weakly infected with either HIV-Wt (distinct accumulation at the cell contact site), HIV-Env-Tr712 or HIV-ΔEnv (both diffuse cytoplasmic staining) as indicated. Dye-labelled cells, which were not visualised by our confocal microcope, are marked with × in the merge. B: Predominant CA localisation patterns in conjugates of weakly infected MT-4 (p) cells (CA accumulation at multiple sites at the cell periphery). Note that due to enhanced per cell CA expression levels in MT-4 cells, exposure times for taking the micrographs in B were approximately half as long as those employed in A in order to allow detection of individual CA clusters in both cases. C. The percentages of HIV-Wt conjugates exhibiting CA accumulation at the contact site (in the case of permissive MT-4 cells with or without accumulation elsewhere at the cell periphery) was set at 100% and the percentages of HIV-Env-Tr712 and HIV-ΔEnv conjugates exhibiting this phenotype calculated relative to this. The mean percentages from several experiments in weakly infected H9 cells (np, low) or MT-4 cells (p, low) are given.

It has been reported that during cell-to-cell HIV transmission, HIV-Gag protein accumulates at the VS and that this is reduced in the absence of Env [46]. Since the Env-CT has been shown to be able to influence Gag localisation in other cell systems [28,29], we were interested in comparing the localisation of HIV-Gag in Wt and mutant virion infected H9 (np) and MT-4 (p) cells. For this, cultures were weakly infected (to about 10%) with VSV-G pseudotyped HIV-Wt, HIV-Env-Tr712 or, as negative control, HIV-ΔEnv virions. 48 hours p.i., cell conjugates with non-infected cells were allowed to form and subsequently stained for HIV-CA and cellular F-actin. Analysis by confocal microscopy indicated that both in cultures of permissive or non-permissive cells, the absolute number of contacts was similar when using only uninfected cells as compared to mixtures of infected donors with uninfected targets. Furthermore, no major differences in the frequency of conjugation with uninfected target cells between HIV-Wt-, HIV-Env-Tr712- and HIV-ΔEnv-infected cells, irrespective of the permissivity status of the donor cell were observed (data not shown). This suggests that, at any rate in this cell system, conjugate formation is not necessarily driven by Env interaction with its cognate cellular receptor on the target cell. Polarisation of F-actin to cell-to-cell contacts was observed in some but not all cases, a variability that again did not correlate with cell permissivity or the Env variant used. In contrast, a large fraction (30-60% depending on the experiment) of H9 (np) cell conjugates infected with HIV-Wt displayed a marked accumulation of CA at the contact site (Fig. 5A, left panel) that was distinct from the diffuse cytoplasmic distribution observed in unconjugated cells (not shown). This result is in line with reports on the rapid polarization of the VS following contact formation between donor and target cell [7,14]. Notably, this polarisation of CA to cell-to-cell contacts was significantly reduced in conjugates with HIV-Env-Tr712- and HIV-ΔEnv-infected H9 cells and exhibited a similar diffuse cytoplasmic distribution as observed in unconjugated cells (Fig. 5A, middle and right panels). Quantification revealed that these reductions were to 43% and 51%, respectively, of HIV-Wt that was set to 100% (Fig. 5C, left panel). These findings presented here for HIV-1 are remarkably similar to a recent report on murine leukemia virus (MuLV) for which it has been demonstrated that polarised assembly at cell-cell contacts and release, but not cell conjugation, are mediated by the cytoplasmic tail of MuLV-Env [47].

For both, MuLV and HIV-1, the exact manner in which Env-Wt, via its Env-CT domain, mediates Gag accumulation at the VS remains unclear. It would however appear likely that direct or indirect interaction between the Env-CT and the Gag precursor, Pr55^gag^, could be an obvious manner in which the Env-CT achieves this altered CA (Gag) localisation. A simple hypothesis would thus be that the CT region of Env protein, interacting with CD4 and coreceptor at the VS, undergoes inherent or induced interaction with proximal Gag (MA) and results in Gag localisation and assembly at that site. Unfortunately, using the fixation procedures described here, we were not able to convincingly and reproducibly stain Env protein in conjugates. As an alternative to a direct or indirect physical interaction to the Env-CT being the sole basis for Gag accumulation at the VS, it is also conceivable that localisation of the Env-CT domain to the VS could affect signal transduction processes which, in turn, could mediate preferential Gag transport to that site. Future mechanistic studies are warranted to distinguish between these different models.

It is likely that the increased CA accumulation observed with HIV-Wt reflects the formation of functional VSs capable of transmitting virus. It is, however, notable that even in cultures of cells infected with HIV-Env-Tr712 or HIV-ΔEnv, quite a high percentage of conjugates (15-25%) still exhibited Gag accumulation at the VS. At least in the case of Env-Tr712 these conjugates obviously represent cell contacts which do not mediate efficient viral transfer despite high local concentrations of Gag and the presence of a fusion competent Env. This could mean that the Env-CT may play roles in cell-to-cell transmission beyond the recruitment of Gag. It will be of interest to address whether host cell factors known to interact with the Env-CT, such as the recently described heterodimer of prohibitin 1 and 2 [48], are involved in this activity.

Finally, we assessed the localization of CA in conjugates of permissive MT-4 donor cells weakly infected with HIV-Wt or mutant virus (Fig. 5B). Due to the increased per cell levels of CA expression relative to non-permissive H9 cells, shorter exposure times were employed for this analysis in order to allow detection of individual CA clusters. In general, CA accumulated more in multiple unpolarised plasma membrane patches and less diffusely in the cytoplasm than in H9 cells. CA accumulation at the cell-cell contact sites (generally with additional peripheral patches) was detected in approximately 50% of the conjugates. However, there was no statistically significant reduction in the cases of HIV-Env-Tr712 and HIV-ΔEnv conjugates in comparison to HIV-Wt (Fig. 5C, right panel). It is tempting to postulate that it is the observed increased Gag expression levels in permissive cells which results in both increased CA accumulation at the cell periphery and increased cell-to-cell transmission leading to the Env-CT being dispensable for these processes in this cell type.

## Conclusions

This study reveals a critical role of the HIV-Env-CT for virus spread in cultures of non-permissive cells by both cell-free and cell-to-cell transmission routes. This involvement in HIV-1 transmission correlates with a requirement of HIV-Env-CT for Gag accumulation at the VS and is overcome when donor cells have been infected with saturating amounts of HIV-1. HIV-Env-CT is dispensable for virus transmission and Gag polarization in permissive cells, which inherently exhibit higher HIV gene expression levels. Thus, intracellular concentrations of Gag may dictate to which extent HIV-1 spread depends on the Env-CT.

## Competing interests

The authors declare that they have no competing interests.

## Authors' contributions

VB, VE and OTF designed the study, VE, TP and CH performed the analyses and VB, VE and OTF made contributions with drafting the manuscript.
